# The role of p-Stat3 Y705 immunohistochemistry in glioblastoma prognosis

**DOI:** 10.1186/s13000-019-0903-4

**Published:** 2019-11-05

**Authors:** Sergiu Susman, Radu Pîrlog, Daniel Leucuța, Andrei Otto Mitre, Vlad Adrian Padurean, Carmen Melincovici, Ioana Moldovan, Doinița Crișan, Stefan Ioan Florian

**Affiliations:** 1 Department of Morphological Sciences, Iuliu Hațieganu University of Medicine and Pharmacy, 6 Pasteur Street, 400349 Cluj-Napoca, Romania; 2 Department of Pathology, Imogen Research Centre, Cluj-Napoca, Romania; 3 Department of Medical Informatics and Biostatistics, Iuliu Hațieganu University of Medicine and Pharmacy, Cluj-Napoca, Romania; 4 Department of Neurosurgery, Iuliu Hațieganu University of Medicine and Pharmacy, Cluj-Napoca, Romania; 5 Department of Neurosurgery, Emergency County Hospital, Cluj-Napoca, Romania; 6 Department of Pathology, Emergency County Hospital, Cluj-Napoca, Romania

**Keywords:** Glioblastoma, Stat3, Immunohistochemistry, Prognosis

## Abstract

**Background:**

In spite of the multimodal treatment used today, glioblastoma is still the most aggressive and lethal cerebral tumour. To increase survival in these patients, novel therapeutic targets must be discovered. Signal transducer and activator of transcription 3 (Stat3), a transcription factor that controls normal cell differentiation and survival is also involved in neoplastic celltransformation. In this study we evaluated the immunohistochemical expression of pY705-Stat3 in patients with primary glioblastoma and determined its prognostic role by correlating it with survival.

**Methods:**

This retrospective study included 94 patients diagnosed with glioblastoma. We determined the localization, number of positive cells, and marker intensity for pY705-Stat3 in these patients with the use of immunohistochemistry. The prognostic role was determined by correlating pY705-Stat3 expression on formalin-fixed paraffin-embedded tumour tissues with the patient’s survival in univariate and multivariate COX regressions.

**Results:**

We found a statistically significant difference in survival between the patients with more than 20% pY705-Stat3 positive cells and those with less than 20% pY705-Stat3 positive cells (8.9 months median survival versus 13.7 months medial survival, *p* <  0.001). On multivariate analyses with the COX proportional hazards regression model including pY705-Stat3 expression, age and relapse status, pY705-Stat3 status was an independent prognostic factor in glioblastoma (*P* <  0.001).

**Conclusion:**

The results obtained show that the immunohistochemical expression of pY705-Stat3 correlates with survival in glioblastoma. This study identifies Stat3 as a possible target for existing or new developed Stat3 inhibitors.

## Background

According to the 2016 WHO classification, glioblastoma is classified as a grade IV, being known to be one of the most lethal brain tumours [1]. Standard treatment includes surgical resection followed by adjuvant radiotherapy and chemotherapy [2]. Despite the multimodal treatment used at present, median survival remains very low, and new therapies are required to increase survival in these patients [3].

From a histopathological point of view, glioblastoma is characterized by necrosis and microvascular proliferation, elements that reflect an intra-tumour heterogeneity in terms of cell adaption to hypoxia [1].

Adaptation to hypoxia leads to the specific activation of the genetic programs and signalling pathways that are also involved in the stem-like phenotype or epithelial-mesenchymal (EMT) transition, with the subsequent increase in resistance to chemotherapy and radiotherapy [4–6]. Recent studies that have attempted to decode the molecular mechanisms involved in hypoxic cell adaptation in glioblastoma have shown that the major signalling pathways activated in hypoxia are those of the hypoxic inducible factor 1α (HIF-1α) vascular endothelial growth factor (VEGF), transforming growth factor beta (TGFβ), mammalian target of rapamycin (mTOR), CD133, as well as of signal transducer and activator of transcription 3 (Stat3) [6–9].

Stat3 protein is a regulator of cell survival, proliferation, differentiation and apoptosis; it also regulates cell adaptation to hypoxia by stimulating angiogenesis in several types of cancers [6–11]. As a transcription factor, Stat3 is activated by phosphorylation; after dimerization it translocates into the nucleus, where it binds to the DNA, activating specific cellular pathways [12].

Elevated levels of Stat3 have been highlighted in various types of cancer, including thyroid cancer, melanoma, prostate cancer, Hodgkins lymphoma, breast cancer, hepatocellular carcinoma and glioblastoma [11, 13]. Being involved in numerous signalling pathways and over-expressed in tumour cells compared to normal tissues, Stat3 is considered to be a potential target for future antitumor therapies [10, 12].

In vitro studies have demonstrated the role of Stat3 in glial tumour biology, especially in glioblastoma, being directly involved in maintaining the stem-like phenotype, EMT (epithelial-mesenchymal transition), resistance to alkylating agents such as temozolomide (TMZ) and radiotherapy [5, 14, 15]. Furthermore, in vitro inhibition of Stat3 activation in glioblastoma resulted in a decrease in the proliferative capacity and an increase in cellular sensitivity to TMZ treatment [10, 15, 16]. Although there a wide a range of in vitro studies showing the importance of Stat3 in glioblastoma, there are only a few clinically oriented studies that correlate Stat3 expression with patient survival [17–19].

In this study, we evaluated the prognostic role of the immunohistochemical expression of p-Stat3Y705 in a series of primary glioblastoma patients. In addition, we attempted to establish a threshold for the immunohistochemical expression of this marker that has prognostic significance.

## Methods

### Patients

This retrospective study included 94 patients diagnosed with glioblastoma. Formalin-fixed paraffin-embedded tissue samples were obtained from the archive of the Emergency County Hospital Pathology Department in Cluj-Napoca, Romania. The patients were operated in the period 2009–2015 in the Neurosurgery Department of the same institution. All patients underwent the standard postoperative treatment (chemotherapy and radiotherapy). Patients were regularly checked according to standard protocols. The overall survival time was measured from the time of the first diagnosis of glioblastoma until death.

The diagnosis of glioblastoma was confirmed by two experienced pathologists (SS and DC) in line with the current WHO classification system [1]. We examined the medical record of each patient to determine age, gender, tumour volume (calculated by the product of the three diameters of the tumor divided by 2 [20], tumour localization, relapse and survival.

The study was conducted with the approval of the Ethics Committee of the Iuliu Haţieganu University of Medicine and Pharmacy Cluj-Napoca, Romania.

### Immunohistochemistry

Immunohistochemistry was performed automatically on 3-μm-thick sections of formalin-fixed and paraffin-embedded tumour specimens with DAKO Omnis®, using the ethylenediaminetetraacetic acid (EDTA), at pH = 9, for antigen retrieval. For the immunohistochemical assessment of Stat3 expression, the specimens were incubated overnight at 4 °C with Phospho-Stat3 (Tyr705) antibody, clone D3A7 (Cell Signalling Technology, Inc., Danvers, MA) at a 1:400 dilution. Positive and negative controls were used in line with the recommendation of the manufacturer. We assessed the percentage of Stat3 positive tumour cells (0–100%) and immunostaining intensity (absent, poor, moderate or intense). For all cases the area of necrosis was assessed by morphometry, using an Olympus BX 46 microscope. Images were taken with an Olympus UC 30 camera system with UI52 with an infinity correction optical system and processed in the LabSense 1.2 Olympus Software by GmbH Imaging Solutions.

The slides were read independently by two experienced pathologists with no knowledge of clinical data. In the case of divergent results, the slides were reviewed by both pathologists working together, and consensus was reached. The K score for the two pathologists that analysed the specimens was 0.81.

### Statistical analysis

Categorical data were presented as absolute and relative frequencies. Continuous data that did not follow the normal distribution were presented as the median and interquartile range (IQR). Overall survival (OS) was defined as the period from the primary surgery until death of the patient. We considered a 20% positivity of tumour cells as a cut-off for STAT3 immunopositivity, which respected the proportional hazard assumption for the Cox regression. The cut-off point was initially set to > 0, > 5%, and then increased to 10%, and then 20%. We increased the cut-off points because the proportional hazard assumption was not met for > 0, 5, 10%, but it was met for 20%. Comparisons between groups in respect of survival were performed using the long-rank test, and presented with a Kaplan Meier plot. The STAT3 immunopositivity was assessed univariately and then in a multivariate model (adjusting for age and tumour localization) using Cox proportional hazards regression analysis. We checked the proportional hazard assumption with a formal test. Results were expressed as a hazard ratio and its corresponding 95% confidence interval. A two tailed *p*-value less than 0.05 was considered as statistically significant. All analyses were performed in R environment for statistical computing and graphics, version 3.2.3.

## Results

Table 1 presents the characteristics of the patients included in our study by age, gender, tumour volume, location and clinical evolution (death and relapse).

**Table 1:** Patient characteristics

Characteristic	N (%) (Total = 94)
Age (yr.) median (IQR)	51.5 (44–56)
Gender	
M	53 (56.4)
F	41 (43.6)
Deceased	88 (93.62)
Relapse	24 (25.5)
Tumor volume (cm^3^) median (IQR)	23.26 (10.35–44.81)
Localization	
Left	37 (39.4)
Right	38 (40.4)
Bilateral	6 (6.4)
n.a.	13 (13.8)
Frontal	45 (47.87)
Parietal	22 (23.4)
Temporal	28 (29.79)
Occipital	12 (12.77)
Brainstem	2 (2.13)
Other	11 (11.7)

Regarding localization, the tumours were mainly located in the frontal, parietal, temporal and occipital lobes. Other locations included: brainstem, cerebellum, pineal zone, basal ganglia, corpus callosum. All patients received multimodal treatment (surgery followed by radiotherapy and chemotherapy). Data regarding the actual treatment received were available for 53 patients (44 received both radiotherapy and chemotherapy, and three received radiotherapy). The doses were 75 mg/m^2^ for TMZ, and 60 Gy in 15 fractions for the radiotherapy.

### Stat3 immunohistochemistry

**Fig. 1:**
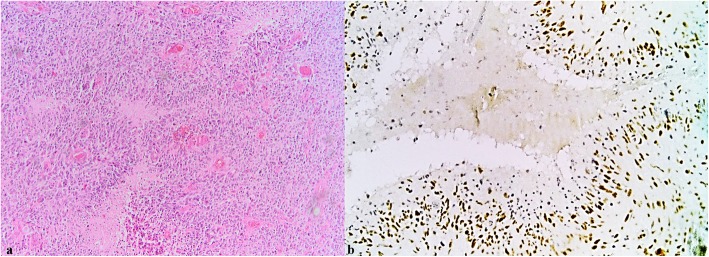
**a** Necrosis is one of the histologic hallmarks of glioblastoma. Necrotic areas are usually seen in a serpentine or geographic pattern. Tumour cells migrate away creating a moving wave of palisading cells around the necrotic areas. (HE 10X) **b** Immunohistochemical expression of p-Stat3Y705 at the nuclear level. The cells are positive mainly in areas with hypoxia located around the necrosis (20X)

**Fig. 2:**
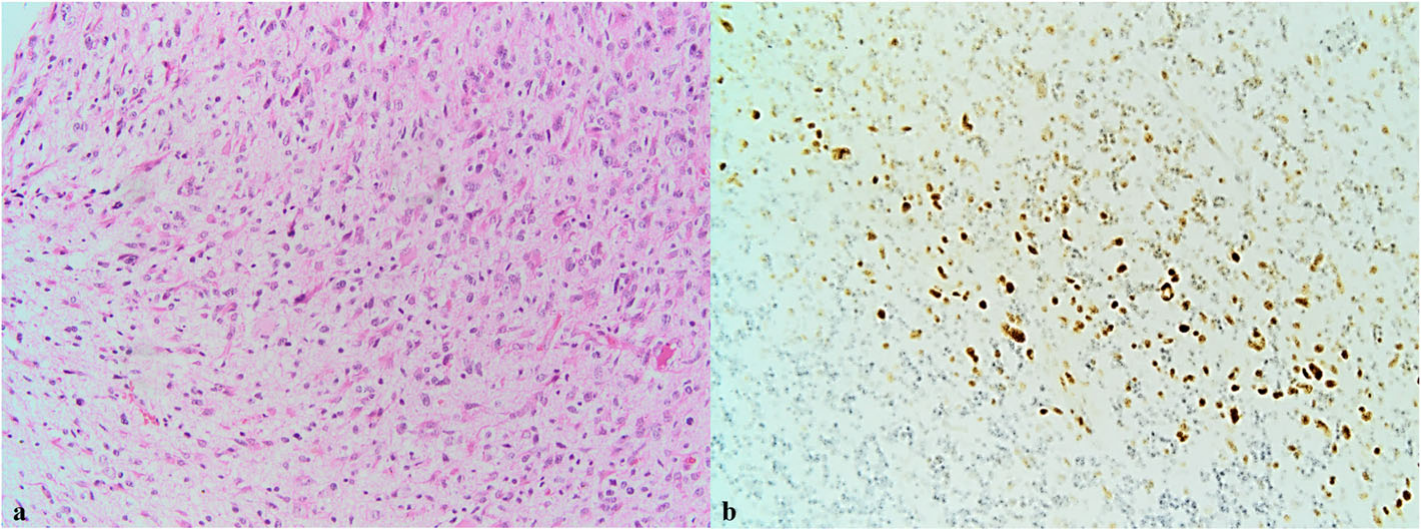
**a** Tumour cells dispersed in the normal brain (HE 20X). **b** Immunohistochemical expression for p-Stat3Y705 at the tumour invasion front. We can evidence an increased intensity of expression where the tumour invades the normal brain tissue (20X)

**Fig. 3:**
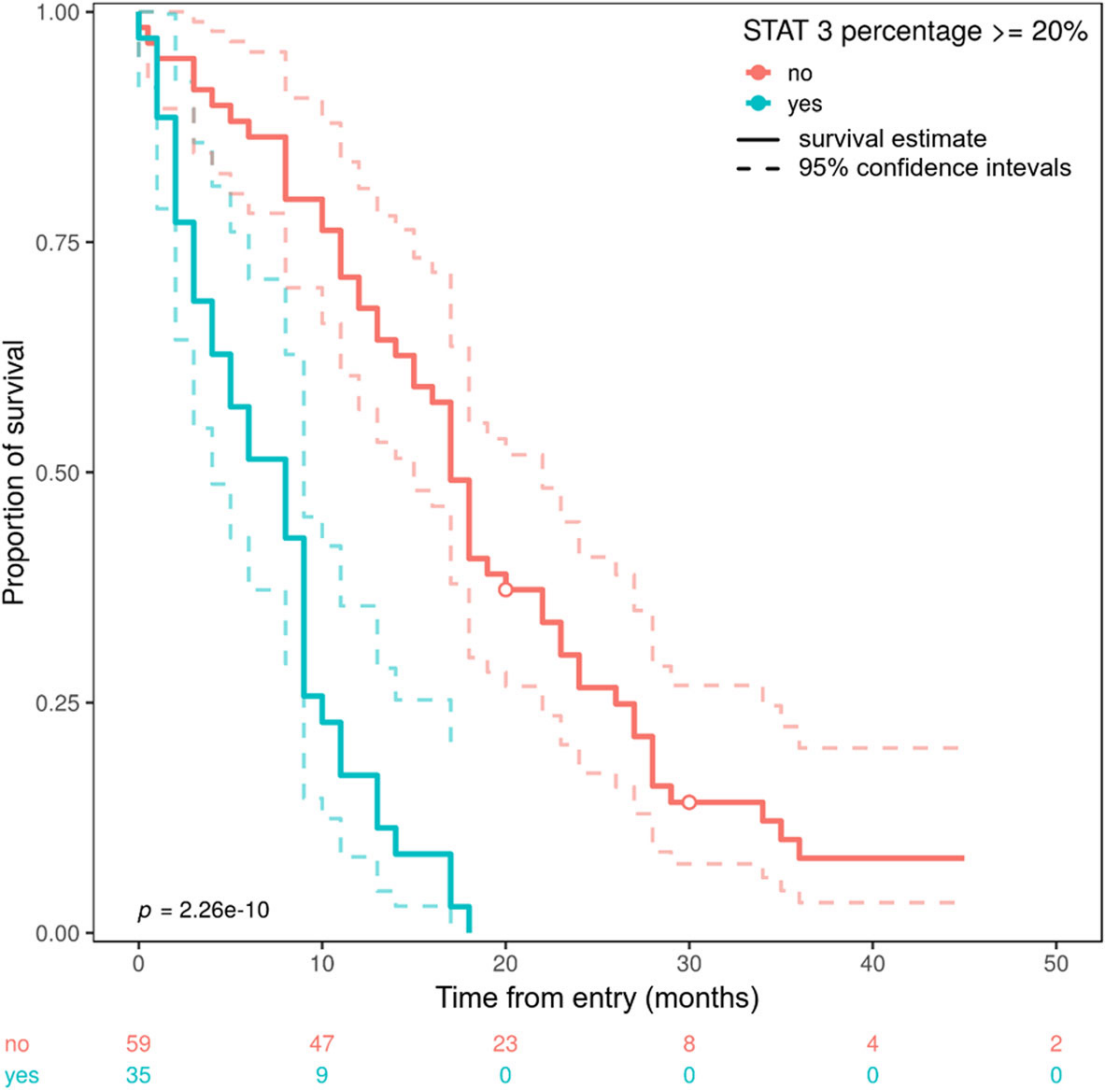
Cumulative overall survival of 94 patients with glioblastoma comparing subjects with less than 20% p-Stat3Y705 positive cells and those with more than 20% positive cells

We found p-Stat3 Y705 immunopositive cells in 89% of our glioblastoma cases. The percentage of p-Stat3 Y705 positive cells was low, with less than 5% positive cells in 18 (19.78%) cases, intermediate, with positive cells between 5 and 9% in 23 (25.27%) cases, and high, with more than 10% Stat3 positive cells in 50 (54.95%) cases. The intensity of the nuclear staining was absent in 10 (11%), poor in 43 (47.3%), moderate in 36 (39.6%) and intense in 2 (2.2%) of the cases. To assess the immunohistochemical marker, the entire tumour surface was analysed. This has the advantage of highlighting the number of positive cells, the intensity of the marker, and the distribution of positive cells across the entire tumour surface. In our series, we could observe heterogeneity, the cells being positive for p-Stat3 Y705 at the nuclear level mainly in the perinecrotic areas and at the tumour invasion front (Figs. 1 and 2).

p-Stat3 Y705 immunohistochemical expression influences survival in glioblastoma. We found a statistically significant survival difference between subjects with more than 20% p-Stat3 Y705 positive cells (17 months median follow-up), who had better survival rates than those with less than 20% positive cells (8 months median follow-up), *p* < 0.001, log-rank test (Fig. 3).

The same observation holds true in the Cox proportional hazards regression (HR) model in univariate and then in multivariate analyses, including p-Stat3 Y705 expression status (> 20%) even after adjusting for age and different tumour localization (Table 2). Age remains an important survival predictor. Localization was not associated with survival, and only parietal localization was close to the statistically significant level as a predictor factor. Furthermore, we created other models to adjust for other prognostic factors besides p-Stat3 expression (Table 3). Thus, we added gender, tumour volume, and necrosis, and p-Stat3 Y705 expression status was an independent prognostic factor in glioblastoma (*P* <  0.001). None of the adjusted factors was statistically significant associated with survival, except age and parietal localization.

**Table 2:** Univariate and multivariate Cox proportional hazard regression models predicting overall survival

	Univariable			Multivariable		
	HR	(95% CI)	*p*-value	HR	(95% CI)	*p*-value
Age (years)	1.02	(1–1.05)	0.039	1.04	(1.01–1.06)	0.01
p-Stat3 percentage ≥ 20%	4.86	(2.91–8.11)	< 0.001	5.69	(3.27–9.91)	< 0.001
Frontal	0.84	(0.55–1.28)	0.419	1.02	(0.61–1.71)	0.945
Parietal	0.86	(0.52–1.41)	0.555	0.6	(0.35–1.04)	0.068
Temporal	0.94	(0.59–1.49)	0.799	0.84	(0.5–1.42)	0.522
Occipital	0.81	(0.44–1.5)	0.511	0.81	(0.4–1.64)	0.562
Brainstem	1.24	(0.3–5.07)	0.764	2.91	(0.62–13.75)	0.177

The multivariate model includes all the variables in the table

A second model was created adding to these variables, gender, as well as tumour volume (cm^3^), where we found HR p-Stat3 percentage ≥ 20% = 4.86 (95% CI 2.91–8.11), *p* <  0.001

*HR* Hazard ratio, *CI* Confidence interval

**Table 3:** Multivariate Cox proportional hazard regression models predicting overall survival, for p-Stat3 percentage adjusting for different confounders

Models	HR	(95% CI)	*P*-value
Model 1	5.69	(3.27–9.91)	< 0.001
Model 2	4.86	CI 2.91–8.11	< 0.001
Model 3	7.8	(2.88–21.11)	< 0.001

Model 1 - multivariate Cox proportional hazard regression model including age (years), Stat3 percentage > = 20%, localizations (frontal, parietal, temporal, occipital, brainstem)

Model 2 – model 1 plus gender and tumour volume (cm^3^)

Model 3 – model 2 plus necrosis (μm^2^)

*HR* Hazard ratio, *CI* Confidence interval

## Discussion

In this study, we evaluated the p-Stat3y705 expression in a group of primary glioblastomas by immunohistochemistry and correlated the expression with the overall survival. In our study, an increased number of immunopositive p-Stat3y705 cells correlated with a reduced overall survival. Our data are in line with those of previous studies reporting that an increase of Stat3 level associates with a poor prognosis in glioblastoma patients, highlighting the clinical value of Stat3 evaluation for patients with glioblastoma [17–19]. These results support the importance of a routine immunohistochemical evaluation of the phosphorylation status of p-Stat3y705 as a prognostic marker of clinical interest for patients with glioblastoma regardless of age or relapse status.

It is important to note that by evaluating the whole tumour surface, we observed a specific localization for Stat3 immunopositivity in cells that surround the necrotic areas, but also in cells located at the tumour invasion front. This suggests the presence of an intra-tumoural heterogeneity of cells in terms of the activated intracellular signalling pathways and could account for the resistance of a subset of cells to the current treatment, and the high relapse rate.

The perinecrotic localization of p-Stat3y705 immunopositive cells suggests the activation of the signalling pathways involved in cellular adaptation to hypoxia, Stat3 being a potent activator of HIF-1α, and VEGF [19, 21]. HIF-1 can also be activated by mTOR proteins, either directly or via the STAT3 pathway [22, 23]. HIF-1 is known to be involved in stimulating tumour motility and radio resistance to current therapies, explained by the formation of “palisades” due to an outward migration of tumour cells toward the more vascularised zones [19, 24, 25]. VEGF can be up-regulated either by Stat3 and/or by HIF-1. Through its expression, it promotes the activation of neoangiogenesis in glioblastoma, highlighting the role of Stat3 in cellular adaptation in extreme conditions [7, 21, 26]. Furthermore, Stat3 is an activator of the epithelial-mesenchymal transition (EMT) process in glioblastoma, both through TGFβ and HIF-1α, thus enhancing tumour high motility, invasiveness and chemo resistance, which could account for the positivity of cells at the invasion front [27–30]. HIF-1 and Stat3 can also activate the transcription of zinc finger transcription factor (ZEB) proteins, especially ZEB1, which promotes EMT, migration and invasion [31, 32]. Stat3 activation in the invasion front cells could be an important observation. After surgical resection, these cells dispersed in the normal brain are at the origin of tumour recurrence. Developing inhibitors that target the signalling pathways activated specifically in cells at the tumour invasion front could lead to a more effective postoperative therapy.

Glioma stem cells (GSC) are a subtype of cells found in glioblastoma that are involved in tumour recurrence, initiation and invasiveness [33]. GSC are also believed to be involved in tumour chemo- and radio resistance to therapy [34]. CD133, a surface protein, is a known marker for GSC, its expression being correlated with tumour malignancy [23]. In low oxygenated cells, HIF-1α, mTOR, TGFβ and Stat3 levels were shown to increase CD133 expression in tumour cells, promoting GSC formation and proliferation [6, 9, 14, 35, 36]. Altogether, hypoxia and Stat3 are involved in promoting glioblastoma stem-like cells, thus increasing tumour resistance and invasiveness [37–39].

O6-methylguanyl DNA methyltransferase (MGMT) is an enzyme that helps repair the damaged DNA sequence in glioblastoma, preventing alkylating chemotherapic agents such as TMZ to be fully efficient [40]. Epigenetic hypermethylation of the MGMT promoter silences the gene predicting a favourable response to alkylating chemotherapy in glioblastoma patients [41, 42]. Therefore, MGMT promoter methylation status can be used as a marker for resistance to treatment with alkylating agents, especially in elderly patients, where chemotherapy in un-methylated tumours evidenced minimal benefit [40, 43]. Stat3 expression is required for MGMT activity, and inhibition of Stat3 leads to a down-regulation of MGMT, favouring TMZ therapy [16].

Regarding the in vitro studies that have evaluated Stat3 inhibition in GMB, Han el al showed that Cpd188 is able to enhance the effect of chemo-radiotherapy treatment for the cell lines with a p-Stat overexpression [44]. Another study has provided data regarding molecular mechanisms underlying hypoxia-induced glioma cell autophagy, evidencing that the inhibition of IL6-Stat3 axis represses autophagy in glioblastoma cell lines in vitro [45]. The same study has demonstrated a certain level of efficiency by blocking Il6 receptor with tocilizumab. Given that Stat3 is situated at the interconnection of signalling pathways that controls the cellular processes involved in hypoxia, resistance to treatment, EMT, the stem-like phenotype and migration, it could be interesting to consider STAT3 inhibitors as a potentially therapeutic approach in glioblastoma treatment [15, 16, 46].

Stat3 inhibitors are currently an important issue, as early clinical data has shown promising results in EGFR mutation- positive non-small cell lung cancer. However, more research is required to understand the different mechanisms that lead to the activation of STAT3 in order to design robust Stat3-targeted therapies [15]. Another example of inhibitors targeting Stat3 at different levels and currently used in clinical trials are: STAT3 antisense oligonucleotide (advanced stage/metastatic hepatocellular carcinoma) or STAT3 SH2 domain binder (multiple myeloma, non-Hodgkin lymphoma, acute myeloid leukaemia, chronic myeloid leukaemia) [47]. Overall, these data could open the perspective for future clinical trials using Stat3 inhibitors in glioblastoma.

As with any observational study, potential confounders that were not taken into account could influence the observed results. We used multivariate analysis and we adjusted for several variables, but we didn’t include the different treatment timing/dosages.

## Conclusion

Our results show that Stat3 expression can be evaluated by immunohistochemistry and has an important prognostic role in glioblastoma. Therefore, clinical approaches should consider including a Stat3 inhibitor in the therapeutic protocol in order to improve tumour response to radiotherapy and chemotherapy. Furthermore, another observation of our study is the preferred localization of Stat3 positive cells at the nuclear level of tumour cells in the perinecrotic areas and at the tumour invasion front. This discovery could suggest the presence of heterogeneous cell populations that are involved in tumour development and treatment resistance. In order to better understand the malignant potential of glioblastomas, we should consider glioblastoma consists of distinct cell subpopulations. This approach could uncover molecular contexts of susceptibility that will accelerate the development of new therapies targeting cells that matter.

## Data Availability

The datasets used and/or analysed during the current study are available from the corresponding author on reasonable request.

## Abbreviations

CI: Confidence interval
EDTA: Ethylenediaminetetraacetic acid
EMT: Epithelial-mesenchymal transition
GSC: Glioma stem cells
HE: Hematoxylin-Eozin
HIF-1α: Hypoxic inducible factor 1α
HR: Hazard regression
IQR: Interquartile range
MGMT: O6-methylguanyl DNA methyltransferase
mTOR: Mammalian target of rapamycin
OS: Overall survival
Stat3: Signal transducer and activator of transcription 3
TGFβ: Transforming growth factor beta
TMZ: Temozolomide
VEGF: Vascular endothelial growth factor
yr.: Years
ZEB: Zinc finger transcription factor

## References

[1] Louis DN, Perry A, Reifenberger G, von Deimling A, Figarella-Branger D, Cavenee WK (2016). The 2016 World Health Organization classification of tumors of the central nervous system: a summary. Acta Neuropathol.

[2] Wen PY, Kesari S (2008). Malignant gliomas in adults. N Engl J Med.

[3] Stupp R, Mason WP, van den Bent MJ, Weller M, Fisher B, Taphoorn MJB (2005). Radiotherapy plus concomitant and adjuvant temozolomide for glioblastoma. N Engl J Med.

[4] Kubelt C, Hattermann K, Sebens S, Mehdorn HM, Held-Feindt J (2015). Epithelial-to-mesenchymal transition in paired human primary and recurrent glioblastomas. Int J Oncol.

[5] Kahlert UD, Nikkhah G, Maciaczyk J (2013). Epithelial-to-mesenchymal(−like) transition as a relevant molecular event in malignant gliomas. Cancer Lett.

[6] Platet N, Liu SY, El Atifi M, Oliver L, Vallette FM, Berger F (2007). Influence of oxygen tension on CD133 phenotype in human glioma cell cultures. Cancer Lett.

[7] Yokogami K, Yamashita S, Takeshima H (2013). Hypoxia-induced decreases in SOCS3 increase STAT3 activation and upregulate VEGF gene expression. Brain Tumor Pathol.

[8] Fukumura D, Xu L, Chen Y, Gohongi T, Seed B, Jain RK (2001). Hypoxia and acidosis independently up-regulate vascular endothelial growth factor transcription in brain tumors in vivo. Cancer Res.

[9] Matsumoto K, Arao T, Tanaka K, Kaneda H, Kudo K, Fujita Y (2009). mTOR signal and hypoxia-inducible factor-1 regulate CD133 expression in cancer cells. Cancer Res.

[10] Kim JE, Patel M, Ruzevick J, Jackson CM, Lim M (2014). STAT3 activation in glioblastoma: biochemical and therapeutic implications. Cancers (Basel).

[11] Bromberg J (2002). Stat proteins and oncogenesis. J Clin Invest.

[12] Gray GK, McFarland BC, Nozell SE, Benveniste EN. NF-kB and STAT3 in glioblastoma: therapeutic targets coming of age. Expert Rev Neurother. 2014;14. 10.1586/14737175.2014.964211. 10.1586/14737175.2014.964211 PMC4350260 25262780

[13] Zuo M, Li C, Lin J, Javle M (2015). LLL12, a novel small inhibitor targeting STAT3 for hepatocellular carcinoma therapy. Oncotarget.

[14] Kim E, Kim M, Woo D-H, Shin Y, Shin J, Chang N (2013). Phosphorylation of EZH2 activates STAT3 signaling via STAT3 methylation and promotes tumorigenicity of glioblastoma stem-like cells. Cancer Cell.

[15] Wong ALA, Hirpara JL, Pervaiz S, Eu J-Q, Sethi G, Goh B-C (2017). Do STAT3 inhibitors have potential in the future for cancer therapy?. Expert Opin Investig Drugs.

[16] Kohsaka S, Wang L, Yachi K, Mahabir R, Narita T, Itoh T (2012). STAT3 inhibition overcomes temozolomide resistance in glioblastoma by downregulating MGMT expression. Mol Cancer Ther.

[17] Lin G-S, Chen Y-P, Lin Z-X, Wang X-F, Zheng Z-Q, Chen L (2014). STAT3 serine 727 phosphorylation influences clinical outcome in glioblastoma. Int J Clin Exp Pathol.

[18] Birner P, Toumangelova-Uzeir K, Natchev S, Guentchev M (2010). STAT3 tyrosine phosphorylation influences survival in glioblastoma. J Neuro-Oncol.

[19] Rodrigues BR, Queiroz-Hazarbassanov N, Lopes MH, Bleggi-Torres LF, Suzuki S, Cunha IW (2016). Nuclear unphosphorylated STAT3 correlates with a worse prognosis in human glioblastoma. Pathol Res Pract.

[20] Sreenivasan S, Madhugiri V, Sasidharan G, Kumar RR (2016). Measuring glioma volumes: a comparison of linear measurement based formulae with the manual image segmentation technique. J Cancer Res Ther.

[21] Xu Q, Briggs J, Park S, Niu G, Kortylewski M, Zhang S (2005). Targeting Stat3 blocks both HIF-1 and VEGF expression induced by multiple oncogenic growth signaling pathways. Oncogene.

[22] Huang W-J, Chen W-W, Zhang X (2016). Glioblastoma multiforme: effect of hypoxia and hypoxia inducible factors on therapeutic approaches. Oncol Lett.

[23] Dodd KM, Yang J, Shen MH, Sampson JR, Tee AR (2015). mTORC1 drives HIF-1α and VEGF-A signalling via multiple mechanisms involving 4E-BP1, S6K1 and STAT3. Oncogene.

[24] Brat DJ, Castellano-Sanchez AA, Hunter SB, Pecot M, Cohen C, Hammond EH (2004). Pseudopalisades in glioblastoma are hypoxic, express extracellular matrix proteases, and are formed by an actively migrating cell population. Cancer Res.

[25] Rong Y, Durden DL, Van Meir EG, Brat DJ, "Pseudopalisading" necrosis in glioblastoma: a familiar morphologic feature that links vascular pathology, hypoxia, and angiogenesis. J Neuropathol Exp Neurol. 2006;65:529-39. 10.1097/00005072-200606000-00001 16783163

[26] Spence AM, Muzi M, Swanson KR, O’Sullivan F, Rockhill JK, Rajendran JG (2008). Regional hypoxia in glioblastoma multiforme quantified with [18F] fluoromisonidazole positron emission tomography before radiotherapy: correlation with time to progression and survival. Clin Cancer Res.

[27] Lo H-W, Cao X, Zhu H, Ali-Osman F (2008). Constitutively activated STAT3 frequently coexpresses with epidermal growth factor receptor in high-grade gliomas and targeting STAT3 sensitizes them to Iressa and alkylators. Clin Cancer Res.

[28] Liu R-Y, Zeng Y, Lei Z, Wang L, Yang H, Liu Z (2014). JAK/STAT3 signaling is required for TGF-β-induced epithelial-mesenchymal transition in lung cancer cells. Int J Oncol.

[29] Herrera-Perez RM, Voytik-Harbin SL, Sarkaria JN, Pollok KE, Fishel ML, Rickus JL (2018). Presence of stromal cells in a bioengineered tumor microenvironment alters glioblastoma migration and response to STAT3 inhibition. PLoS One.

[30] Cruickshanks N, Zhang Y, Hine S, Gibert M, Yuan F, Oxford M (2019). Discovery and therapeutic exploitation of mechanisms of resistance to MET inhibitors in glioblastoma. Clin Cancer Res.

[31] Jhanwar-Uniyal M, Labagnara M, Friedman M, Kwasnicki A, Murali R (2015). Glioblastoma: molecular pathways, stem cells and therapeutic targets. Cancers (Basel).

[32] Chou C-C, Chuang H-C, Salunke SB, Kulp SK, Chen C-S (2015). A novel HIF-1α-integrin-linked kinase regulatory loop that facilitates hypoxia-induced HIF-1α expression and epithelial-mesenchymal transition in cancer cells. Oncotarget.

[33] Zhang W, Shi X, Peng Y, Wu M, Zhang P, Xie R, et al. HIF-1α promotes epithelial-mesenchymal transition and metastasis through direct regulation of ZEB1 in colorectal cancer. PLoS One. 2015;10. 10.1371/journal.pone.0129603. 10.1371/journal.pone.0129603 PMC4461314 26057751

[34] Chen K, Huang Y, Chen J (2013). Understanding and targeting cancer stem cells: therapeutic implications and challenges. Acta Pharmacol Sin.

[35] Zorzan M, Giordan E, Redaelli M, Caretta A, Mucignat-Caretta C (2015). Molecular targets in glioblastoma. Future Oncol.

[36] You H, Ding W, Rountree CB (2010). Epigenetic regulation of cancer stem cell marker CD133 by transforming growth factor-β. Hepatology.

[37] Peñuelas S, Anido J, Prieto-Sánchez RM, Folch G, Barba I, Cuartas I (2009). TGF-β increases glioma-initiating cell self-renewal through the induction of LIF in human glioblastoma. Cancer Cell.

[38] Zhang C, Mukherjee S, Tucker-Burden C, Ross JL, Chau MJ, Kong J (2017). TRIM8 regulates stemness in glioblastoma through PIAS3-STAT3. Mol Oncol.

[39] Ganguly D, Sims M, Cai C, Fan M, Pfeffer LM (2018). Chromatin remodeling factor BRG1 regulates stemness and chemosensitivity of glioma initiating cells. Stem Cells.

[40] Guryanova OA, Wu Q, Cheng L, Lathia JD, Huang Z, Yang J (2011). Nonreceptor tyrosine kinase BMX maintains self-renewal and tumorigenic potential of glioblastoma stem cells by activating STAT3. Cancer Cell.

[41] Wick W, Weller M, van den Bent M, Sanson M, Weiler M, von Deimling A (2014). MGMT testing—the challenges for biomarker-based glioma treatment. Nat Rev Neurol.

[42] Crespo I, Vital AL, Gonzalez-Tablas M, Patino Mdel C, Otero A, Lopes MC (2015). Molecular and genomic alterations in glioblastoma multiforme. Am J Pathol.

[43] Hegi ME, Diserens A-C, Gorlia T, Hamou M-F, de Tribolet N, Weller M (2005). *MGMT* gene silencing and benefit from temozolomide in glioblastoma. N Engl J Med.

[44] Han TJ, Cho BJ, Choi EJ, Kim DH, Song SH, Paek SH (2016). Inhibition of STAT3 enhances the radiosensitizing effect of temozolomide in glioblastoma cells in vitro and in vivo. J Neuro-Oncol.

[45] Xue H, Yuan G, Guo X, Liu Q, Zhang J, Gao X (2016). A novel tumor-promoting mechanism of IL6 and the therapeutic efficacy of tocilizumab: hypoxia-induced IL6 is a potent autophagy initiator in glioblastoma via the p-STAT3-MIR155-3p-CREBRF pathway. Autophagy.

[46] Haftchenary S, Luchman HA, Jouk AO, Veloso AJ, Page BDG, Cheng XR (2013). Potent targeting of the STAT3 protein in brain cancer stem cells: a promising route for treating glioblastoma. ACS Med Chem Lett.

[47] Johnson DE, O’Keefe RA, Grandis JR (2018). Targeting the IL-6/JAK/STAT3 signalling axis in cancer. Nat Rev Clin Oncol.

